# Post-traumatic stress symptom trajectories following exposure to population-level trauma: findings from the COVID-19 Healthcare Staff Wellbeing Survey

**DOI:** 10.1192/bjo.2026.11027

**Published:** 2026-04-27

**Authors:** Kevin F. W. Dyer, Niamh Hurst, Ciaran Shannon, Julie-Ann Jordan

**Affiliations:** School of Psychology, Queen‘s University Belfast, UK; IMPACT Research Centre, Northern Health and Social Care Trust, Antrim, UK; Psychological Therapies Service, Northern Health and Social Care Trust, Antrim, UK

**Keywords:** Post-traumatic stress, PTSD, COVID-19, pandemic, healthcare staff

## Abstract

**Background:**

The COVID-19 pandemic has been described as a prolonged societal trauma providing new understanding of long-term post-traumatic stress reactions, both generally and in specific at-risk populations.

**Aims:**

The present study examined the longitudinal course of post-traumatic stress disorder (PTSD) symptoms within one of the most high-profile risk groups (i.e. healthcare staff).

**Method:**

The sample comprised 439 healthcare staff who completed the Northern Ireland longitudinal COVID-19 Staff Wellbeing Survey on a minimum of 3 out of 4 distribution time points. The survey was administered repeatedly over 4 years, spanning both peri- and post-pandemic periods (2020–2023), and contained the Impact of Event Scale-Revised, as well as bespoke items on COVID-19, demographics, occupational issues and support factors.

**Results:**

Three distinct classes emerged from a three-class, latent class growth analysis model. A ‘resilient’ group (74%) displayed symptoms that remained below cut-offs for clinically significant moderate–severe post-traumatic stress throughout the pandemic, whereas a ‘recovering’ group (23%) exhibited moderate–severe symptoms during the pandemic, which then decreased to subthreshold levels post-pandemic. A key at-risk group was the ‘chronic’ class (4%), which had moderate–severe post-traumatic stress symptoms peri-pandemic that continued to increase post-pandemic. Significant predictors of the ‘recovering’ and ‘chronic’ classes included perception of poor communication within the healthcare organisation; increased exposure to COVID-19 outside their work; and increased personal health risk factors for COVID-19.

**Conclusions:**

Post-pandemic PTSD monitoring and support for healthcare staff may be warranted alongside the development of internal communication strategies within healthcare systems to protect staff and services going forward.

The COVID-19 pandemic emerged as a unique societal trauma that had a substantive impact on the psychological well-being of the global population.^1^ Clinically high levels of anxiety-depression (21%) and post-traumatic stress (17%) were reported in the general population during the pandemic.^2^ Recent studies estimate that, whereas there has been a drop-off in mental health difficulties from peak levels, post-pandemic rates remain elevated compared with pre-COVID-19.^3,4^ Such cross-sectional measurements can be misleading, however, because the long-term course of mental health symptoms has been evidenced as non-uniform, with variation across specific groups within the wider population.^5^

Researchers conducting longitudinal studies have observed notable trends. López-Castro et al^6^ assessed post-traumatic stress disorder (PTSD) levels in the general public at four time points between April 2020 and July 2021. Four groups emerged with different symptom trajectories across the measurement points, specifically (a) a large subgroup that did not develop clinical levels of PTSD (73%, ‘resilient’ group); (b) a subgroup with clinical levels of PTSD at baseline that decreased to subclinical levels midway before increasing to clinical levels at final measurement (13.3%, ‘recurring’ group); (c) a subgroup with clinical levels of PTSD at baseline that decreased to subclinical levels at final measurement (8.3%, ‘recovering’ group); and (d) a subgroup with clinical levels of PTSD at baseline that were maintained at final measurement (5.5%, ‘chronic’ group).

Despite the explanatory utility of the findings, this study has several methodological limitations, including the fact that participants were not required to rate their PTSD symptoms using COVID-19 as the index trauma. Instead, participants could rate symptoms in relation to any potentially traumatic event they had experienced (e.g. physical assault). Moreover, the longitudinal measurements were taken during only the first year of the pandemic and did not extend to post-pandemic, which makes it questionable to draw conclusions about post-traumatic reactions following a societal trauma. Shevlin et al^1^ corrected for the first of these issues, anchoring symptom ratings to COVID-19 in their study and measuring across five time points during the first year of the pandemic. That investigation found five classes, including three that were comparable to the groups of López-Castro et al:^6^ ‘resilient’, ‘recovering’ and ‘chronic’ (i.e. ‘resilient’ (67.7%), ‘adaptive’ (8.4%) and ‘chronic’ (4.9%), respectively), but with two additional classes. These were (a) a ‘moderate-stable’ subgroup that had consistently moderate clinical levels across time points; and (b) a ‘deteriorating’ subgroup with subclinical levels of PTSD at baseline that deteriorated across the pandemic to clinically high levels at final measurement.^1^

Although the studies cited thus far provide key insights into the general long-term course of PTSD, as well as post-COVID-19 trauma responses, it is widely acknowledged that during the pandemic several subpopulations were at increased risk of post-traumatic reactions. Higher levels of clinically significant symptoms were reported in healthcare workers on anxiety (27%), depression (36%) and PTSD (32%) compared with the wider population. Explanations for these heightened symptoms include enhanced exposure to psychological and organisational stressors in healthcare staff job roles.^7,8^ Jordan et al^9^ used longitudinal data from four peri-pandemic time points spanning November 2020 to August 2021, and identified two groups of healthcare workers with different PTSD symptom trajectories. Eighty-four per cent of staff had post-traumatic stress symptoms that fluctuated in line with COVID-19 in-patient levels but remained at subclinical levels, whereas a substantial minority (16%) had persistent clinical levels of PTSD symptoms across all phases of the pandemic.

## Aims

The present study examined the longitudinal course of PTSD symptoms within healthcare staff both during and after the COVID-19 pandemic. Considering that healthcare staff were a vulnerable population during the outbreak and now play a pivotal role in societal recovery from COVID-19, it was deemed important to identify at-risk groups who may have developed debilitating PTSD trajectories. The investigation followed on from Jordan et al,^9^ and used latent class growth analysis (LCGA) to examine the trajectories of post-traumatic stress symptoms reported by healthcare staff over a 2-year period (February 2021 to May 2023). In line with the three trajectories identified in studies reported by López-Castro et al^6^ and Shevlin et al,^1^ it was predicted that the majority of healthcare staff would maintain subthreshold levels of post-traumatic stress symptoms peri- and post-pandemic (i.e. ‘resilient’ group), whereas a minority of healthcare staff would develop clinically significant levels of symptoms that would either resolve post-pandemic (i.e. ‘recovering’ group) or continue at these higher levels (i.e. ‘chronic’ group). It was also hypothesised that personal, demographic and organisational stressors would predict membership of post-traumatic stress trajectory classes (i.e. ‘recovering’ and ‘chronic’ groups).

## Method

### Participants

All health and social care staff in Northern Ireland were eligible to take part in the COVID-19 Staff Wellbeing Survey, which was administered at five time points spanning both the peri- and post-pandemic periods. The present study used data from 4 of the 5 survey time points: Time 2 (T2., 8–28 February 2021; *n* = 2898); T3 (10–30 May 2021; *n* = 2480); T4 (9–29 August 2021; *n* = 2119); and T5 (15 May–12 June 2023; *n* = 2047). T2–4 occurred during the pandemic, and T5 coincided with the declaration by the World Health Organization in May 2023 that the COVID-19 global health emergency was over.^10,11^ In Northern Ireland, T5 also occurred simultaneously with the collection of many COVID-19 health statistics being discontinued following a sustained and substantive reduction in deaths caused by COVID-19.^12^

The survey was advertised to staff via broadcast emails, health service social media, screensavers and laminated posters in staff areas. Data from the four time points were merged using work or personal email addresses, which was an optional response field in the survey. Analyses were conducted on data reported by a sample of 439 healthcare workers who participated and provided their email addresses on at least 3 of the 4 time points. The demographic profile of the longitudinal sample is shown in Table 1. The gender and age profile of the sample is consistent with Northern Ireland Health and Social Care Workforce census data.^13^

**Table 1:** Demographic characteristics of the sample at Time 2

Variables	Score Time 2	Northern Ireland Health and Social Care Workforce census, %^13^
Age, years, mean (s.d.)	45.65 (9.99)	NA
≤ 34, frequency (%)	68 (15)	28
35–44, frequency (%)	116 (26)	26
45–54, frequency (%)	158 (36)	27
≥ 55, frequency (%)	97 (22)	19
Gender, frequency (%)		
Male	87 (20)	21
Female	352 (80)	79
Occupation, frequency (%)		
Nursing and midwifery	77 (18)	33
Administrative and clerical	150 (34)	19
Medical	24 (6)	7
Professional and technical	117 (27)	15
Social services	49 (11)	13
Other	22 (5)	13

NA, not available.

For the longitudinal sample, Time 3 data for gender and occupation were used if Time 2 data were missing.

Participants in occupational groups with desk-based roles, and hence with greater access to computers at work to facilitate completion of an online survey, tended to be over-represented in the sample (administrative and clerical Time 2 34% *v*. population data 19%; professional and technical Time 2 27% *v*. population data 15%^13^). By contrast, occupations such as nursing and midwifery (T2 18% *v*. population data 34%) were underrepresented.

### Measures

#### Post-traumatic stress symptoms

The 22-item Impact of Event Scale-Revised^14^ was used to assess post-traumatic stress severity. The index trauma for ratings was anchored to COVID-19, with participants reporting symptoms relating to the COVID-19 outbreak within the past 7 days at T2–5. Participants were specifically instructed to ‘indicate how distressing each difficulty has been for you DURING THE PAST SEVEN DAYS with respect to the COVID-19 outbreak’. Responses to the items on this DSM-IV-compatible measure were assessed using 5-point Likert items (0 = not at all; 4 = extremely; scale range 0–88). A cut-off of ≥26 was used to indicate clinically significant moderate-to-severe symptoms of post-traumatic stress symptoms, in line with other international studies on healthcare staff well-being during COVID-19.^15^

#### Predictor variables

A range of demographic, occupational and support variables as measured at T2 were considered as potential predictors of post-traumatic stress class membership. Demographic variables included gender (male/female); age (years); a measure of exposure to COVID-19 (scale range 0–7, higher scores being indicative of greater exposure); and if they had at least 1 of 10 COVID-19 risk factors (coding 0 = no; 1 = yes) listed in the COVID-19 Pandemic Mental Health Questionnaire^16^ (e.g. diabetes, chronic liver disease).

Occupational information recorded included whether they had managed patients with COVID-19 (0 = no; 1 = yes); whether their employer had asked them to consider a redeployment opportunity during COVID-19 (0 = no; 1 = yes); and the perceived effectiveness of communication from their organisation on COVID-19-related matters (0 = not effective; 4 = very effective). The demographic and occupational variables are described in full in Jordan et al.^7^

Participants were shown a range of team supports (e.g. Schwartz rounds, ‘buddy’ system) and asked how many were available to them during the last 3 months of the pandemic. Similarly, a range of staff supports (e.g. staff well-being helpline, drop-in centre) were listed, and participants indicated which of these they had used in these last 3 months. From this, 4 variables were constructed: whether at least 1 form of team supports was available (0 = no; 1 = yes); whether they had used at least 1 form of staff supports (0 = no; 1 = yes); total number of team supports available (0–8); and total number of staff supports used (0–9).

### Procedure

Health- and social care workers were invited to take part in the survey online via the Survey Mechanics platform. Participants indicated consent at each time point by clicking to start the questionnaire once they had read the participant information sheet, which provided full details of the study and of their rights as participants.

The authors assert that all procedures contributing to this work comply with the ethical standards of the relevant national and institutional committees on human experimentation, and with the Helsinki Declaration of 1975 as revised in 2013. All procedures involving human subjects/patients were approved by the West of Scotland Research Ethics Service (reference 20/WS/0122, 26 August 2020).

### Statistical analysis

The data were prepared for analysis in IBM SPSS version 30 for Windows (IBM, Armonk, New York, USA; https://www.ibm.com/products/spss), then transferred to Mplus version 8.8 for Windows (Mplus, Los Angeles, California, USA; https://www.statmodel.com/programs.shtml)^17^ for inferential analyses. Missing data rates by time point for the longitudinal sample were as follows: T2 (12%), T3 (6%), T4 (15%) and T5 (44%). Logistic regression was used to assess whether demographic, occupational and pandemic-related T2 variables (e.g. gender; age; perceived effectiveness of communication regarding COVID-19; exposure to COVID-19; awareness of available supports) predicted drop-out at T5. Analysis revealed that being female and having lower awareness of available organisational staff well-being supports, as measured at T2, significantly predicted drop-out at T5. Missing values in the unconditional growth curve models and unconditional latent class growth analysis were dealt with using full-information, maximum-likelihood estimation. Initially, a series of unconditional growth curve models of varying shape (linear, quadratic and free time scores) were fitted to the T2–5 post-traumatic stress scores, and fit was evaluated with reference to acceptable range guidelines.^18^ The freed loading model provided the best fit to the data (*x* ^2^(3) = 5.51, *P* = 0.138; comparative fit index 1.00; Tucker–Lewis index 0.99; root mean square error of approximation 0.04; standardised root mean residual 0.02). In the freed loading model, T2 was coded as 0 and T5 as 1, meaning that growth rate represented change between T2 and 5.

Missing data on T2 predictor variables were dealt with via multiple imputation using the Markov chain Monte Carlo method to generate 20 data-sets, under the assumption of missing at random. Significant predictors of missingness at T2 were included in the models, alongside the outcome variable and T3–5 versions of the T2 predictor variables, because these were strongly related. There was significant variation in both intercept and slope (Table 2) parameters, indicating the possible existence of subpopulations.^19^ A series of LCGA models with 2–5 classes specified were then fitted to the post-traumatic stress T2–5 data. Fit statistics and class trajectory plots were inspected to determine the number of classes that provided best fit to the data.^20–23^ It was determined that a three-class model provided the optimal solution to the data (Table 3). Rates of missingness across time points varied among the classes ‘chronic’ (T2 = 13%, T3 = 5%, T4 = 15%, T5 = 42%), ‘recovering’ (T2 = 6%, T3 = 13%, T4 = 0%, T5 = 63%) and ‘resilient’ (T2 = 9%, T3 = 9%, T4 = 15%, T5 = 46%).

**Table 2:** Unconditional growth curve model (freed loading) for post-traumatic stress

Variables	Post-traumatic stress score (s.e.)
Intercept	18.10 (0.81); *P* < 0.001
Slope	−8.58 (0.85); *P* < 0.001
Variance (intercept)	257.40 (24.64); *P* < 0.001
Variance (slope)	205.50 (37.47); *P* < 0.001
*R* ^a^	−154.71 (25.93); *P* < 0.001

a Intercept with slope covariance.

**Table 3:** Fit indices for unconditional growth mixture models with one to five classes

Fit indices	1-class	2-class	3-class	4-class	5-class
Post-traumatic stress scores					
AIC	11 611.18	11 189.42	11 044.20	10 980.17	10 884.83
BIC	11 643.86	11 234.35	11 101.38	11 049.61	10 966.52
SABIC	11 618.47	11 199.44	11 056.95	10 995.66	10 903.04
Entropy	–	0.89	0.91	0.88	0.87
LMR-LRT	–	0.007	0.005	0.627	0.002
BS-LRT	–	<0.001	<0.001	<0.001	<0.001

AIC, Akaike information criterion; BIC, Bayesian information criterion; SABIC, sample-size adjusted BIC; LMR-LRT, Lo–Mendell–Rubin likelihood ratio test; BS-LRT, bootstrapped likelihood ratio test.

Adopting the three-step manual approach,^24^ predictors were then incorporated into the models. Initially a series of univariate multinomial logistic regression models were run to assess whether demographic (gender, age, exposure to COVID-19, having at least one COVID-19 risk factor), organisational (managed patients with COVID-19, perceived effectiveness of communication regarding COVID-19, whether asked to consider being redeployed) and support variables (whether team supports were available, whether used staff well-being supports, number of team supports available, number of staff well-being supports used) predicted class membership. The significant predictors (exposure to COVID-19, having at least one COVID-19 risk factor, perceived effectiveness of communication regarding COVID-19) were then entered simultaneously into a multinomial logistic regression model.

## Results

A 3-class model provided the best fit for post-traumatic stress symptom T2–5 scores (Table 3).

### Post-traumatic stress trajectories

Three distinct classes were evident from the three-class LCGA model. Whereas Akaike information criterion, Bayesian information criterion (BIC) and sample-size adjusted BIC continued to decrease throughout classes 1–5, scree plots^25^ revealed that no further substantive decreases occurred after 3 classes. Entropy was highest for 3 classes, and the average latent class probabilities for classes 2 and 3 were all in the ‘ideal’ range (>0.90^26^), in contrast to the class 4 and 5 models, which included multiple values in the ‘acceptable’ range. Lo–Mendell–Rubin likelihood ratio test suggested that a three-class solution provided a better fit than a two-class, but no significant improvement for a four-class solution compared with a three-class.

**Fig. 1:**
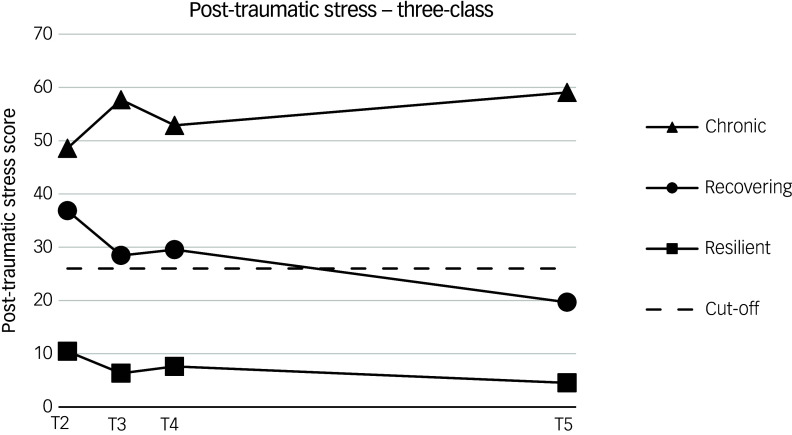
Trajectories of the three-class, post-traumatic stress, latent class growth analysis model. T, time. Time is scaled to account for unequal intervals of measurement.

The means of the three classes weighted by estimated class probability are shown in Fig. 1, and intercept and slope (T2–5 change) parameter estimates are displayed in Table 4. The Impact of Event Scale-Revised cut-off score is also overlain, and indicates the threshold of clinically significant moderate–severe post-traumatic stress symptom severity, rather than a formal PTSD diagnosis. The largest class, ‘resilient’ (*n* = 324; 74%), is characterised by mean symptom scores consistently below the cut-off and a general decreasing trend over time, except for a small increase at T4 that corresponded with an increase in COVID-19 hospital activity in Northern Ireland relative to T3. The ‘recovering’ class (*n* = 99; 23%) also displayed mean levels that fluctuated in line with the peaks and troughs of the pandemic. By contrast, the ‘recovering’ class started with mean levels well in excess of the cut-off, then displayed symptom reduction over time to the point that symptom levels were actually below the cut-off by T5. The ‘chronic’ class (*n* = 16; 4%) was the smallest, starting with mean levels above the cut-off; however, their symptoms generally worsened over time and were at their highest by T5. In contrast to the other two classes, the ‘chronic’ class self-reported greater symptoms when COVID-19 hospital activity was at its lowest: specifically T3 and, in particular, T5.

**Table 4:** Mean scores for growth parameters in the three-class unconditional model

Growth parameters	Post-traumatic stress symptom class		
	Chronic	Recovering	Resilient
	Mean (s.e.)	Mean (s.e.)	Mean (s.e.)
Intercept	48.81 (2.68)***	37.60 (2.04)***	10.38 (0.98)***
Slope	11.77 (4.99)*	−16.92 (3.42)***	−6.06 (1.22)***

*P < 0.05, ***P < 0.001.

### Prediction of class membership

Table 5 displays the logistic coefficients for predictors of post-traumatic stress symptom class membership.

**Table 5:** Multinomial logistic regression of predictors of class membership

Predictors	Comparison	
	Recovering versus resilient (reference)	Chronic versus resilient (reference)
Exposure to COVID-19	0.34 (0.10)**	0.68 (19)***
Having at least one COVID-19 risk factor	0.62 (0.30)*	1.90 (0.64)**
Perceived effectiveness of communication regarding COVID-19	−0.62 (0.15)***	−0.86 (0.29)**

*P < 0.05, **P < 0.01, ***P < 0.001.

A higher level of exposure to COVID-19, having at least one COVID-19 risk factor and perceiving communication from their organisation to be less effective were all associated with a greater likelihood of being in the ‘recovering’ or ‘chronic’ classes than the ‘resilient’ class.

## Discussion

In line with the study hypotheses, three distinct post-traumatic stress symptom trajectories were observed in healthcare workers during and after the COVID-19 pandemic, specifically (a) a ‘resilient’ group (74%), with symptoms that remained below cut-offs throughout the pandemic; (b) a ‘recovering’ group (23%), with initially moderate-to-severe post-traumatic stress symptoms that decreased to subthreshold levels post pandemic; and (c) a ‘chronic’ group (4%), with initially moderate-to-severe levels of symptoms that continued to increase post-pandemic. These trajectories highlight that the developmental course for the majority of healthcare staff (96%) was either not to develop clinically significant levels of post-traumatic stress symptoms or to recover by the end of the pandemic. Worryingly, however, for the small percentage of individuals (4%) who experienced chronic levels of clinically significant post-traumatic stress, their symptom trajectory implies a continued escalation of symptoms post-pandemic, making them an at-risk group. In contrast to wider anecdotal evidence, a number of factors concerned with healthcare organisational exposure to trauma and availability of staff supports (e.g. managing patients with COVID-19, usage of staff supports) were found not to be related to class membership. Instead, perception of poor communication within the healthcare organisation emerged as the only employment-related factor that predicted membership of both post-traumatic stress symptom classes. Other significant predictors were general risk variables such as increased exposure to COVID-19 in everyday life and personal health risk factors for COVID-19.

The present study found post-traumatic stress trajectories that were broadly similar to a number of those obtained in general population studies examining the longitudinal course of mental health symptoms across the pandemic, namely ‘resilient’, ‘recovering’ and ‘chronic’.^1,6^ Although it is not possible to make direct comparisons among such studies, it nonetheless highlights the fact that the course of PTSD symptoms for healthcare workers seems to be somewhat comparable to that of the wider public, even though the proportions in each class may vary due to specific challenges faced by this population and methodological differences. This finding is perhaps unsurprising, given that healthcare staff differ primarily in regard to the severity of COVID-19 stressors and symptoms, whereas the fundamental patterns of change probably reflect universal traumatological processes that operate similarly across society, regardless of occupation.

The predominant role of poor communication in contributing to COVID-19-related PTSD among both the general public and healthcare staff has received support from several studies.^7,27^ The pandemic is widely acknowledged as having fostered a unique ‘internal communication crisis’ for workplaces.^28^ In such crisis circumstances, employees have both increased anxiety and a greater need for information from their host organisation, with provision of clear, frequent, transparent and useful information important for alleviating distress.^29^ However, the COVID-19 pandemic presented a number of obstacles that made such a response challenging, including the following: (a) healthcare managers were under considerable pressure, which reduced capacity for communication; (b) reliable and timely information was often unavailable due to the evolving nature of the pandemic; and (c) many established forms of communication were no longer viable (e.g. in-person team meetings), with a marked shift towards new methods (e.g. online meetings, newsletters) that proved to be a difficult adjustment.^29,30^

Although the precise role of communication in the trajectories observed requires further exploration, it is likely that challenges in this area, combined with staff members’ unmet need for information in order to alleviate distress, contributed to the trajectories of post-traumatic stress in the ‘chronic’ and ‘recovering’ groups. It is possible that a number of other psychological and employment variables played an important explanatory role, including moral distress and the availability of wider staff supports.^31,32^ For example, the present study identified that individual risk factors, such as increased exposure to COVID-19 in everyday life and personal health risks for COVID-19, were predictive of post-traumatic stress trajectories, suggesting that some individuals may have held perceptions of being more physically vulnerable when working during the pandemic, or of posing a heightened risk of infecting others. Such psychological appraisals would be in line with established PTSD risk factors, including vulnerability beliefs and moral distress.^33,34^

### Clinical implications

Long-term post-pandemic PTSD monitoring and support for the healthcare workforce may be warranted based on the symptom courses observed in the present study, with future studies repeating such symptom measurements. The ‘chronic’ group trajectory indicates an escalating trend in post-traumatic stress reactions at the final post-pandemic assessment point. It is therefore quite possible that these symptoms in the at-risk group may have continued or even become exacerbated as time passed, as documented in other longitudinal studies of chronic PTSD.^5^ Indeed, the 3 post-pandemic trajectories obtained in the present study evolved from 2 peri-pandemic trajectories (i.e. low-symptom class (84%), high-symptom class (16%)) obtained using the same population.^9^ Considering the inherent variability over the course of PTSD,^35^ it could be the case that the other groups, most notably the ‘recovering’ group, had later deviations in their trajectory, potentially returning to clinically significant levels. A number of general PTSD studies have noted significant changes in symptom trajectories in longer-term, 7- to 29-year follow-ups.^5,35^ The risk of relapse is also potentially heightened following further exposure to similar traumatic experiences.^36^ This paints a concerning picture for healthcare workers in the ‘chronic’ and ‘recovering’ groups, who are now likely to be working in healthcare systems under significant pressure following the pandemic (e.g. waiting list backlogs, severe staff shortages^37^) and may be vulnerable to further aversive professional experiences and moral distress.^38^ Consequently, it is vital for the continued analysis of these trajectories, as well as both the empirical validation and development of PTSD interventions for healthcare staff, to offset mental health deterioration in these potentially reactivating circumstances.

The study also has implications for understanding PTSD following societal trauma, in providing support to the relatively consistent finding of ‘resilient’, ‘recovering’ and ‘chronic’ trajectories across populations and following large-scale traumatic events (e.g. warfare, pandemics). Ineffective internal and external communication has also been highlighted as a significant issue in regard to pandemic responses, particularly COVID-19.^28^ The present findings highlight the fact that internal communication can be particularly impactful on the well-being of healthcare staff during and after pandemic response, and therefore effective communications strategies should be researched and developed in the advent of future crises to ensure that the well-being of healthcare staff is preserved.

### Limitations

The present study has a number of strengths and weaknesses. A key strength is the longitudinal design permitting both peri- and post-pandemic follow-up on the course of post-traumatic stress symptoms in a substantive sample of healthcare workers. However, despite the methodological rigour, a number of important analyses were not supportable due to sampling limitations: most notably, the small size of the ‘chronic’ group (4%) meant that statistical power was not available to examine predictors of ‘chronic’ versus ‘recovery’ classes, which would have been useful in understanding the mechanisms of trajectory development. However, because the effects of COVID-19 exposure, COVID-19 risk factors and perceived ineffective communication were consistently stronger for the ‘chronic’ compared with the ‘recovering’ group (when compared with the ‘resilient’ class), it is likely that there is some degree of a dose–response relationship between risk factors and likelihood of being in the ‘chronic’ class. Caution should be exercised when interpreting the predictors of class membership analysis, because statistical power is limited for the gender variable given that males comprise only a small proportion of the sample. As a non-probability sample was used, self-selection bias also cannot be ruled out and it is possible that those with mental health issues were more likely to have engaged in the study across and after the pandemic,^39^ which limits sample representativeness and potentially inflates prevalence. It should also be acknowledged that, although missing data levels at T5 were high, evidence suggests that when multiple imputation models are carefully specified (e.g. auxiliary variables are factored in), as was done in the present study, estimation remains accurate.^40^ Longitudinal pre-pandemic data on the sample were not available and, in order to minimise survey fatigue, it was possible to carry out only one post-pandemic follow-up (i.e. T5). Therefore, the potential impact of prior mental health on likelihood of class membership and the exact shape of change in trajectories between August 2021 and June 2023 could not be examined.

Exposure to additional traumatic events during the peri- and post-pandemic periods was also not assessed in the present study, and thus their potential contribution to longer-term symptom trajectories cannot be entirely ruled out. However, the largely representative nature of the staff sample – and the fact that most demographic and occupational variables did not predict group membership – reduce the likelihood that trauma exposures were disproportionately or systematically concentrated within a single latent trajectory group (e.g. the ‘chronic’ group). Last, whereas brief self-report measures are appropriate for screening post-traumatic stress symptomatology, their use in determining clinical PTSD levels has been shown to inflate caseness relative to clinical interviews, due to their high sensitivity and low specificity.^8^ Nevertheless, conservative interpretations of clinically significant symptom changes can still be made using these instruments, because repeated clinical interviews for PTSD symptoms across five time points would be extremely challenging within the current longitudinal design.

To conclude, three post-traumatic stress symptom trajectories were observed in healthcare workers during and after the COVID-19 pandemic, namely ‘resilient’, ‘recovering’ and ‘chronic’. Although these groups are similar to classes found in the wider population, the escalating trajectory in the ‘chronic’ group and the unique, demanding nature of working in healthcare settings highlight the need for monitoring staff in healthcare services for PTSD symptoms after the pandemic, and offering the workforce psychological support. Prevention research should focus on internal communication enhancement and strategies within healthcare systems, in order to develop robust protective processes in the advent of future health crises.

## Data Availability

The data that support the findings of this study are available from the corresponding author (K.F.W.D.), upon reasonable request. Demographic data will be aggregated in order to protect the identity of the participants.

## References

[1] Shevlin M , Butter S , McBride O , Murphy J , Gibson-Miller J , Hartman TK , et al. Psychological responses to the COVID-19 pandemic are heterogeneous but have stabilised over time: 1 year longitudinal follow-up of the COVID-19 Psychological Research Consortium (C19PRC) study. Psychol Med 2023; 53: 3245–7. 34538283 10.1017/S0033291721004025 PMC8485012

[2] Shevlin M , Butter S , McBride O , Murphy J , Gibson-Miller J , Hartman TK , et al. Refuting the myth of a ‘tsunami’ of mental ill-health in populations affected by COVID-19: evidence that response to the pandemic is heterogeneous, not homogeneous. Psychol Med 2021; 53: 429–37. 33875044 10.1017/S0033291721001665 PMC8111207

[3] Chen Y , Xiong K , Jinfeng L , Jun D , Jiali Z , Xuan J , et al. Trends and factors influencing the mental health of college students in the post-pandemic: four consecutive cross-sectional surveys. Front Psychol 2024; 15: 1387983. 39086428 10.3389/fpsyg.2024.1387983 PMC11288898

[4] Jamshaid S , Bahadar N , Jamshed K , Rashid M , Afzal MI , Tian L , et al. Pre- and post-pandemic (COVID-19) mental health of international students: data from a longitudinal study. Psychol Res Behav Manage 2023; 16: 431–46. 10.2147/PRBM.S395035 PMC9939801 36814636

[5] Solomon Z , Mikulincer M , Ohry A , Ginzburg K. Prior trauma, PTSD long-term trajectories, and risk for PTSD during the COVID-19 pandemic: a 29-year longitudinal study. J Psychiatr Res 2021; 141: 140–5. 34198195 10.1016/j.jpsychires.2021.06.031 PMC9750185

[6] López-Castro T , Papini S , Bauer A , Swarbrick M , Paul LK , Nizzi MC , et al. Posttraumatic stress disorder symptom trajectories in a 16-month COVID-19 pandemic period. J Traumat Stress 2023; 36: 180–92. 10.1002/jts.22899 PMC9880687 36572985

[7] Jordan J-A , Shannon C , Browne D , Carroll E , Maguire J , Kerrigan K , et al. COVID-19 Staff wellbeing survey: longitudinal survey of psychological well-being among health and social care staff in Northern Ireland during the COVID-19 pandemic. BJPsych Open 2021; 7: e159. 34493960 10.1192/bjo.2021.988 PMC8410744

[8] Scott HR , Stevelink SA M , Gafoor R , Lamb D , Carr E , Bakolis I , et al. Prevalence of post-traumatic stress disorder and common mental disorders in health-care workers in England during the COVID-19 pandemic: a two-phase cross-sectional study. Lancet Psychiatry 2022; 10: 40–9. 36502817 10.1016/S2215-0366(22)00375-3 PMC9731576

[9] Jordan J-A , Shannon C , Browne D , Carroll E , Maguire J , Kerrigan K , et al. Healthcare staff mental health trajectories during the COVID-19 pandemic: findings from the COVID-19 Staff Wellbeing Survey. BJPsych Open 2023; 9: e112. 37345555 10.1192/bjo.2023.497 PMC10305016

[10] United Nations. WHO Chief Declares End to COVID-19 as a Global Health Emergency . UN News, 2023 (https://news.un.org/en/story/2023/05/1136367).

[11] Wise J. COVID-19: WHO declares end of global health emergency. BMJ 2023; 381: 1041. 37160309 10.1136/bmj.p1041

[12] Northern Ireland Statistics and Research Agency. *Coronavirus (COVID-19) Statistics*. NISRA, 2025 (https://www.nisra.gov.uk/statistics/people-and-communities/coronavirus-covid-19-statistics).

[13] O’Hagan J. Northern Ireland Health and Social Care Workforce Census March 2020 . Department of Health, 2020 (https://www.health-ni.gov.uk/sites/default/files/publications/health/hscwc-march-20.pdf).

[14] Weiss DS , Marmar CR. The impact of event scale—revised. In Assessing Psychological Trauma and PTSD (eds JP Wilson , TM Keane ): 399–411. The Guilford Press, 1997.

[15] Lai J , Ma S , Wang Y , Cai Z , Hu J , Wei N , et al. Factors associated with mental health outcomes among health care workers exposed to coronavirus disease 2019. JAMA Netw Open 2020; 3: e203976. 32202646 10.1001/jamanetworkopen.2020.3976 PMC7090843

[16] Rek S , Freeman D , Reinhard M , Bühner M , Keeser D , Padberg F. The COVID-19 Pandemic Mental Health Questionnaire (CoPaQ): Introducing a Comprehensive Measure of the Psychosocial Impact of the Coronavirus Crisis . OSF, 2021 (https://osf.io/3evn9/#!). 10.1186/s12888-021-03425-6 PMC8406012 34465319

[17] Muthén LK , Muthén BO. Mplus User‘s Guide 8th ed. Muthén & Muthén, 2017.

[18] Hu L-t , Bentler PM. Cutoff criteria for fit indexes in covariance structure analysis: conventional criteria versus new alternatives. Struct Eq Model 1999; 6: 1–55.

[19] Kandauda W , Lee TK , O’Neal CW , Lorenz F. Higher-order Growth Curves and Mixture Modelling with Mplus: A Practical Guide (Multivariate Applications Series). Routledge, 2016.

[20] Feldman B , Masyn KE , Conger R. New approaches to studying problem behaviours: a comparison of methods for modelling longitudinal, categorical adolescent drinking data. Dev Psychol 2009; 45: 652–76. 19413423 10.1037/a0014851 PMC2791967

[21] Jung T , Wickrama KAS. An introduction to latent class growth analysis and growth mixture modelling. Soc Personal Psychol Compass 2008; 2: 302–17.

[22] Nylund KL , Asparouhov T , Muthén B. Deciding on the number of classes in a latent class analysis and growth mixture modelling: a Monte Carlo simulation study. Struct Eq Model Multidiscipl J 2007; 14: 535–69.

[23] Tein J-Y , Coxe S , Cham H. Statistical power to detect the correct number of classes in latent profile analysis. Struct Eq Model Multidiscipl J 2013; 20: 640–57. 10.1080/10705511.2013.824781 PMC3904803 24489457

[24] Vermunt JK , Magidson J. Latent class cluster analysis. In Advances in Latent Class Analysis (eds JA Hagenaars , AL McCutchen ): 89–106. Cambridge University Press, 2002.

[25] Nylund-Gibson K , Choi AY. Ten frequently asked questions about latent class analysis. Transl Issues Psychol Sci 2018; 4: 440–61.

[26] Weller BE , Bowen NK , Faubert SJ. Latent class analysis: a guide to best practice. J Black Psychol 2020; 46: 287–311.

[27] Wang J , Huang X , Wang Y , Wang M , Xu J , Li X. COVID-19 information overload, negative emotions and posttraumatic stress disorder: a cross-sectional study. Front Psychiatry 2022; 13: 894174. 35693965 10.3389/fpsyt.2022.894174 PMC9186157

[28] Ruck K , Men LR. Guest editorial: internal communication during the COVID-19 pandemic. J Commun Manage 2021; 25: 185–95.

[29] Ecklebe S , Löffler N. A question of quality: perceptions of internal communication during the Covid-19 pandemic in Germany. J Commun Manage 2021; 25: 214–32.

[30] Björk L , Corin L , Akerstrom M , Jonsdottir IH , Innocenti AD , Wijk H , et al. Under pressure – the working situation of Swedish healthcare managers during the first wave of COVID-19. Front Psychol 2023; 13: 1052382. 36710753 10.3389/fpsyg.2022.1052382 PMC9874142

[31] Dyer KFW , Shannon C , McCann L , Mitchell S , Kerrigan K , McClements R , et al. Psychological support for healthcare workers during the COVID-19 pandemic: a mixed methods study involving support providers. Eur J Psychotraumatol 2022; 13: 2151282. 38872604 10.1080/20008066.2022.2151282 PMC9793905

[32] Fattori A , Comotti A , Mazzaracca S , Consonni D , Bordini L , Colombo E , et al. Long-term trajectory and risk factors of healthcare workers mental health during COVID-19 pandemic: a 24 month longitudinal cohort study. Int J Environ Res Public Health 2023; 20: 4586. 36901597 10.3390/ijerph20054586 PMC10002366

[33] Cai W , Ding C , Tang Y-L , Wu S , Yang D. Effects of social supports on posttraumatic stress disorder symptoms: moderating role of perceived safety. Psychol Trauma Theory Res Pract Policy 2014; 6: 724–30.

[34] Norman SB , Feingold JH , Kaye-Kauderer H , Kaplan CA , Hurtado A , Kachadourian L , et al. Moral distress in frontline healthcare workers in the initial epicenter of the COVID-19 pandemic in the United States: relationship to PTSD symptoms, burnout, and psychosocial functioning. Depress Anxiety 2021; 38: 1007–17. 34293236 10.1002/da.23205 PMC8426909

[35] Bonanno GA , Mancini AD , Horton JL , Powell TM , LeardMann CA , Boyko EJ , et al. Trajectories of trauma symptoms and resilience in deployed US military service members: prospective cohort study. Br J Psychiatry 2012; 200: 317–23. 22361018 10.1192/bjp.bp.111.096552

[36] Solomon Z. Combat Stress Reaction: the Enduring Toll of War. Plenum Press, 1993.

[37] British Medical Association. *NHS Backlog Data Analysis*. BMA, 2025 (https://www.bma.org.uk/advice-and-support/nhs-delivery-and-workforce/pressures/nhs-backlog-data-analysis).

[38] Jeffs L , Heeney N , Johnstone J , Hunter J , Loftus CA , Ginty L , et al. Long-term impact of COVID-19 pandemic: moral tensions, distress, and injuries of healthcare workers. PLOS One 2024; 19: e0298615. 39331662 10.1371/journal.pone.0298615 PMC11432829

[39] Eisma MC. Prevalence rates of prolonged grief disorder are overestimated. Eur J Psychotraumatol 2025; 16: 2520634. 40586701 10.1080/20008066.2025.2520634 PMC12210396

[40] Austin PC , van Buuren S. The effect of high prevalence of missing data on estimation of the coefficients of a logistic regression model when using multiple imputation. BMC Med Res Methodol 2022; 22: 196. 35850734 10.1186/s12874-022-01671-0 PMC9290209

